# Rehabilitating homonymous visual field deficits: white matter markers of recovery—stage 1 registered report

**DOI:** 10.1093/braincomms/fcae324

**Published:** 2024-09-21

**Authors:** Hanna E Willis, Matthew R Cavanaugh, Sara Ajina, Franco Pestilli, Marco Tamietto, Krystel R Huxlin, Kate E Watkins, Holly Bridge

**Affiliations:** Wellcome Centre for Integrative Neuroimaging, FMRIB, Nuffield Department of Clinical Neuroscience, University of Oxford, Oxford OX3 9DU, UK; Flaum Eye Institute and Center for Visual Science, University of Rochester, Rochester, NY 14642, USA; Wellcome Centre for Human Neuroimaging, Queen Square Institute of Neurology, University College London, Queen Square, London WC1N 3BG, UK; Department of Psychology, Department of Neuroscience, Center for Perceptual Systems, Center for Learning and Memory, The University of Texas at Austin, Austin, TX 78712-1043, USA; Department of Psychology, University of Torino, Torino 10123, Italy; Department of Medical and Clinical Psychology, Tilburg University, The Netherlands; Flaum Eye Institute and Center for Visual Science, University of Rochester, Rochester, NY 14642, USA; Wellcome Centre for Integrative Neuroimaging, Department of Experimental Psychology, University of Oxford, Oxford OX2 6GG, UK; Wellcome Centre for Integrative Neuroimaging, FMRIB, Nuffield Department of Clinical Neuroscience, University of Oxford, Oxford OX3 9DU, UK

**Keywords:** visual training, stroke, neuroimaging, diffusion-weighted imaging

## Abstract

Damage to the primary visual cortex (V1) or its afferent white matter tracts results in loss of vision in the contralateral visual field that can present as homonymous visual field deficits. Recent evidence suggests that visual training in the blind field can partially reverse blindness at trained locations. However, the efficacy of visual training to improve vision is highly variable across subjects, and the reasons for this are poorly understood. It is likely that variance in residual functional or structural neural circuitry following the insult may underlie the variation among patients. Many patients with visual field deficits retain residual visual processing in their blind field, termed ‘blindsight’, despite a lack of awareness. Previous research indicates that an intact structural and functional connection between the dorsal lateral geniculate nucleus (dLGN) and the human extrastriate visual motion-processing area (hMT+) is necessary for blindsight to occur. We therefore predict that changes in this white matter pathway will underlie improvements in motion discrimination training. Twenty stroke survivors with unilateral, homonymous field defects from retro-geniculate brain lesions will complete 6 months of motion discrimination training at home. Visual training will involve performing two daily sessions of a motion discrimination task, at two non-overlapping locations in the blind field, at least 5 days per week. Motion discrimination and integration thresholds, Humphrey perimetry and structural and diffusion-weighted MRI will be collected pre- and post-training. Changes in fractional anisotropy will be analysed in two visual tracts: (i) between the ipsilesional dLGN and hMT+ and (ii) between the ipsilesional dLGN and V1. The (non-visual) tract between the ventral posterior lateral nucleus of the thalamus (VPL) and the primary somatosensory cortex (S1) will be analysed as a control. Tractographic changes will be compared to improvements in motion discrimination and Humphrey perimetry-derived metrics. We predict that (i) improved motion discrimination performance will be directly related to increased fractional anisotropy in the pathway between ipsilesional dLGN and hMT+ and (ii) improvements in Humphrey perimetry will be related to increased fractional anisotropy in the dLGN-V1 pathway. There should be no relationship between behavioural measures and changes in fractional anisotropy in the VPL-S1 pathway. This study has the potential to lead to greater understanding of the white matter microstructure of pathways underlying the behavioural outcomes resulting from visual training in retro-geniculate strokes. Understanding the neural mechanisms that underlie visual rehabilitation is fundamental to the development of more targeted and thus effective treatments for this underserved patient population.

## Teaser

In this study, Willis *et al.* aim to investigate whether visual training in the blind field can improve visual field deficits post-stroke. Moreover, they will use diffusion-weighted imaging to explore whether changes in white matter microstructure in the dLGN-hMT+ or dLGN-V1 pathway may explain these behavioural improvements.

## Introduction

Retro-geniculate damage to the primary visual cortex (V1) or its immediate afferent tracts causes loss of conscious vision in contralateral portions of the visual field, referred to as homonymous visual field deficits (also know as hemi- or quadrantanopia). This type of vision loss affects between 20 and 57% of stroke survivors and significantly impacts activities of daily living, including mobility, reading and driving as well as quality of life.^1^ A brief 6-month period of spontaneous plasticity exists directly after stroke when visual deficits can improve.^2^ However, in contrast with those suffering from sensorimotor strokes,^3^ occipital stroke survivors are rarely provided with visual rehabilitation; when available, therapies that focus on compensatory eye movement strategies or substitution, such as prism lenses, are usually recommended. The efficacy of such therapies is controversial. A recent Cochrane Review of randomized controlled trials concluded that there was little evidence for the efficacy of current interventions.^1^

A more direct approach to improving vision in visual field deficits is to use training that repeatedly stimulates a portion of the blind field.^4^ These rehabilitation programmes require patients to discriminate visual stimuli within their blind field. They have successfully reduced the size of the visual deficit^5-9^ and improved contrast sensitivity,^8,10^ direction discrimination and orientation discrimination.^10-12^

Despite their success, the efficacy of visual training programmes remains highly variable across participants^5-7,9^ and the reasons for this are poorly understood, although the precise neural mechanisms underlying improvements in vision are yet to be determined. We posit that variability in the extent and location of the stroke damage may help to explain the inconsistency in visual improvement due to the specific fibres that are affected and their potential for plasticity to improve visual function. Here, we will use neuroimaging to measure changes in brain structure in relation to training and recovery of vision in stroke survivors with visual field deficits. The aims of the study are (i) to inform our understanding of the neural mechanisms underlying training-induced visual recovery after occipital stroke and (ii) to determine, based on white matter parameters, which patients are likely to benefit most from this form of training.

Functional training changes the microstructure of white matter pathways and can be quantified using diffusion-weighted imaging. For example, training in the motor system produces increases in fractional anisotropy (FA, a measure of myelination and organization of fibre tracts), which is directly related to improvements in motor skill after training.^13^ In stroke survivors, training increases FA in motor tracts and relates to motor improvement.^14^ FA is predictive of motor outcomes in skill training in healthy controls and stroke survivors.^15,16^ We predict, therefore, that improvements in vision due to training in stroke survivors with visual field deficits will produce measurable FA changes in white matter pathways connecting areas activated by training.

While visual field deficits lead to the loss of normal, conscious vision, some patients retain the ability to detect or discriminate visual information in their blind field. This is known as ‘blindsight’ and has been shown to improve with training.^17^ Studies suggest that intact pathways between the ipsilesional lateral geniculate nucleus (dLGN) and extrastriate motion area (hMT+) are necessary for blindsight.^18-21^ Based on these studies, we predict that increases in FA in the dLGN-hMT+ pathway will underlie training-related improvements in motion discrimination thresholds in visual field deficits (Hypothesis 1).

Blindsight is most reliably elicited by large (>4 degrees), moving stimuli (5–20 Hz).^10^ However, evidence suggests that vision beyond blindsight ability, such as luminance contrast detection, can also be improved by visual training in those with visual field deficits.^6^ Receptive fields in V1 are driven by small spots of light that vary in luminance,^22,23^ such as those used in Humphrey perimetry. Thus, training-induced improvements beyond blindsight abilities may result from bringing functionally impaired, spared V1 back ‘online’.^24,25^ We therefore additionally hypothesize that increases in FA in the dLGN-V1 pathway will underlie training-related improvements in luminance detection as measured by the Humphrey visual fields (HVF; Hypothesis 2).

Twenty participants will undergo visual motion discrimination training inside their blind field for 6 months. MRI scanning using diffusion-weighted imaging before and after training will be used to test the hypotheses that (i) increase in FA in the ipsilesional dLGN-hMT+ pathway will be related to improvement in motion discrimination thresholds and (2) increases in FA in the ipsilesional dLGN-V1 pathway will be related to improvements in luminance detection (HVFs). There should be no relationship between behavioural measures and FA in the VPL-S1 control pathway.

Finally, we will ascertain whether baseline measures of FA in the ipsilesional dLGN-hMT+ pathway can predict visual improvements after 6 months of training (Hypothesis 3).

## Materials and methods

### Ethics and participants

#### Participants

Twenty-four stroke survivors will be recruited (aged 18–80 years; males and females) and will provide written informed consent. Participants will be healthy, MRI-safe, English-speaking adults with damage to V1 sustained in adulthood (18 years+) and resulting in visual field deficits (also known as hemi- or quadrantanopia). They will have suffered damage at least 6 months prior to the study (i.e. they will be in the chronic post-stroke phase). Time since stroke will be recorded for each participant.

Participants recruited for the present study will be required to have no history of diagnosed cognitive or psychiatric disorders, including executive or attentional deficits. Participants will not be included if they have a previously diagnosed executive or attentional deficit or have an impairment that is sufficient to interfere with either testing or training. In addition, recruited participants will have no history of previous eye disease or impairment other than visual field deficits, including all forms of visuospatial neglect. Where there is any suspicion of neglect, we may ask a close relation or friend of potential participants to complete the Catherine Bergego Scale based on their observations.

Participants will not participate in any other visual rehabilitation for the duration of the study. Interviews by phone will be conducted with each participant to determine eligibility for the study. Medical notes may also be provided for this assessment if necessary.

#### Ethics

Ethical approval was given by the local ethics committee (R60132/RE001). Before taking part, participants will have to read the study information sheet and will be made aware that the training to be administered is to inform research and is not an established treatment, nor can it be claimed to guarantee an improvement in vision.

#### Exclusion criteria

Participants will be excluded from the analysis if they (i) develop a neurological, psychiatric or eye disease condition during the study, (ii) are unable to complete the required number of training sessions (minimum 100) within ∼6 months, (iii) are unable to complete the full testing protocol (all pre- and post-training behaviour and diffusion measures), (iv) are unable to fixate during training (see details below) and (v) show fixation losses, false positives and negative errors outside of the normal range (≥20%) in either eye at any time point on the HVFs. We will recruit and enrol 24 participants to account for participants that need to be removed from the analyses. If necessary, we will also recruit further participants to ensure the sample size remains at 20.

#### Power analysis

To the best of our knowledge, there are no previous publications from which we can estimate effect sizes; we therefore computed a sensitivity analysis to determine the effect sizes we will be sensitive to with an achievable sample size. Due to the commitments of the study and rarity of isolated visual field deficits, a sample size of 20 was deemed appropriate. We will use linear regression to determine the relationship between visual improvement and FA change in this study.

A sensitivity power analysis was calculated for a linear regression analysis using the ‘pwr’ package in R^26^ with a sample of size 20, alpha of 0.05 and power of 0.9. From this calculation, we are powered sufficiently to be able to detect large effect sizes (Cohen’s *F* = 0.59) 90% of the time. We will use a one-directional test, as we predict that both behavioural measures and FA will increase with training. In our view, a moderate-to-large effect size is necessary to have the impact on activities of daily living expected from the training and given the duration of the planned intervention. Smaller effect sizes will likely have less real-life significance.

Although there are no-known studies investigating the relationship between FA change and visual improvement in visual field deficits on which to base effect sizes, we are confident that large effect sizes will be achievable in this study. Behavioural training studies in visual field deficits found large or very large effect sizes for within- and between-subject improvements in motion discrimination thresholds (*n* = 17 within-subject *d* = 2.78; *n* = 5 untrained, 17 trained; between-subject *d* = 4.49;^6^ and on HVF measurements.^6,7^

Additionally, studies in the motor cortex report large effect sizes showing significant increases in FA in motor skill training after a 6-week intervention (*n* = 48; partial eta^2^ = 0.17).^13^ Moreover, Fan *et al.*^14^ reported a large effect showing that increased FA in ipsilesional motor tracts in stroke survivors was related to improvements in motor function after a 4-week intervention (*n* = 10; *r* = 0.68). Based on this literature, we therefore predict that we will be sensitive to finding a similarly large effect size in a 6-month intervention in 20 stroke survivors. Thus, according to our sensitivity analysis, we will be well powered (90% power) to investigate the relationship between improvements in vision and change in FA.

### Study design

**Figure 1:**
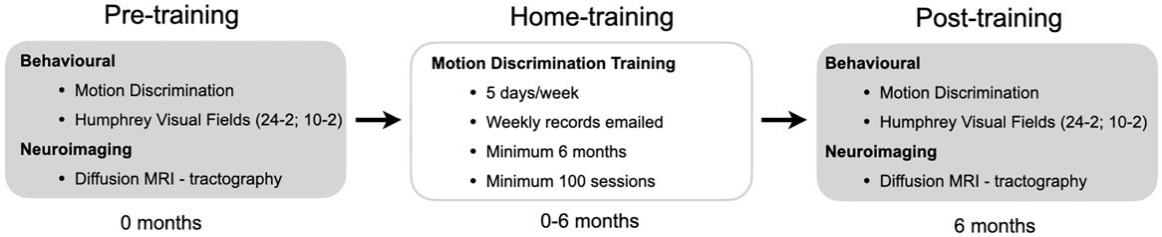
**Overview of rehabilitation protocol.** Study design indicating two research centre visits at pre-training and post-training and 6 months of motion discrimination training to be carried out at home.

Each participant will visit the research centre for assessments on two occasions, pre-training (baseline) and post-training (∼6 months), and complete at-home training between these two time points (see Fig. 1 for overview). At-home training will involve two sessions of visual training (∼40 min total per day), at least 5 days per week for 6 months. On average, participants will complete 150 sessions over a 6-month period, and all participants will complete at least 100 sessions.

### Visual restoration training paradigm

Participants will be trained on a psychophysical, visual training programme.^5-12^ The training programme will be designed in MATLAB (MathWorks) using the Psychophysics Toolbox.^27,28^ Participants will use lab-issued chin-forehead rests and software customized to their own computer and monitor specifications (dimensions, resolution and refresh rate). The programme will be compiled in MATLAB using the Compiler app (MathWorks) and sent to the participant. At the baseline visit, the research team will assist with the set-up of the programme and ensure participants are aware of how to use it appropriately. Each participant will be given instructions detailing how to set the training up at home to ensure consistency. Participants will be encouraged to train when they are most awake, and the exact time will be recorded in the daily logs produced by the training programme. Before training begins, the programme will present a square and participants will be asked to measure the size to ensure the screen dimensions are accurate.

**Figure 2:**
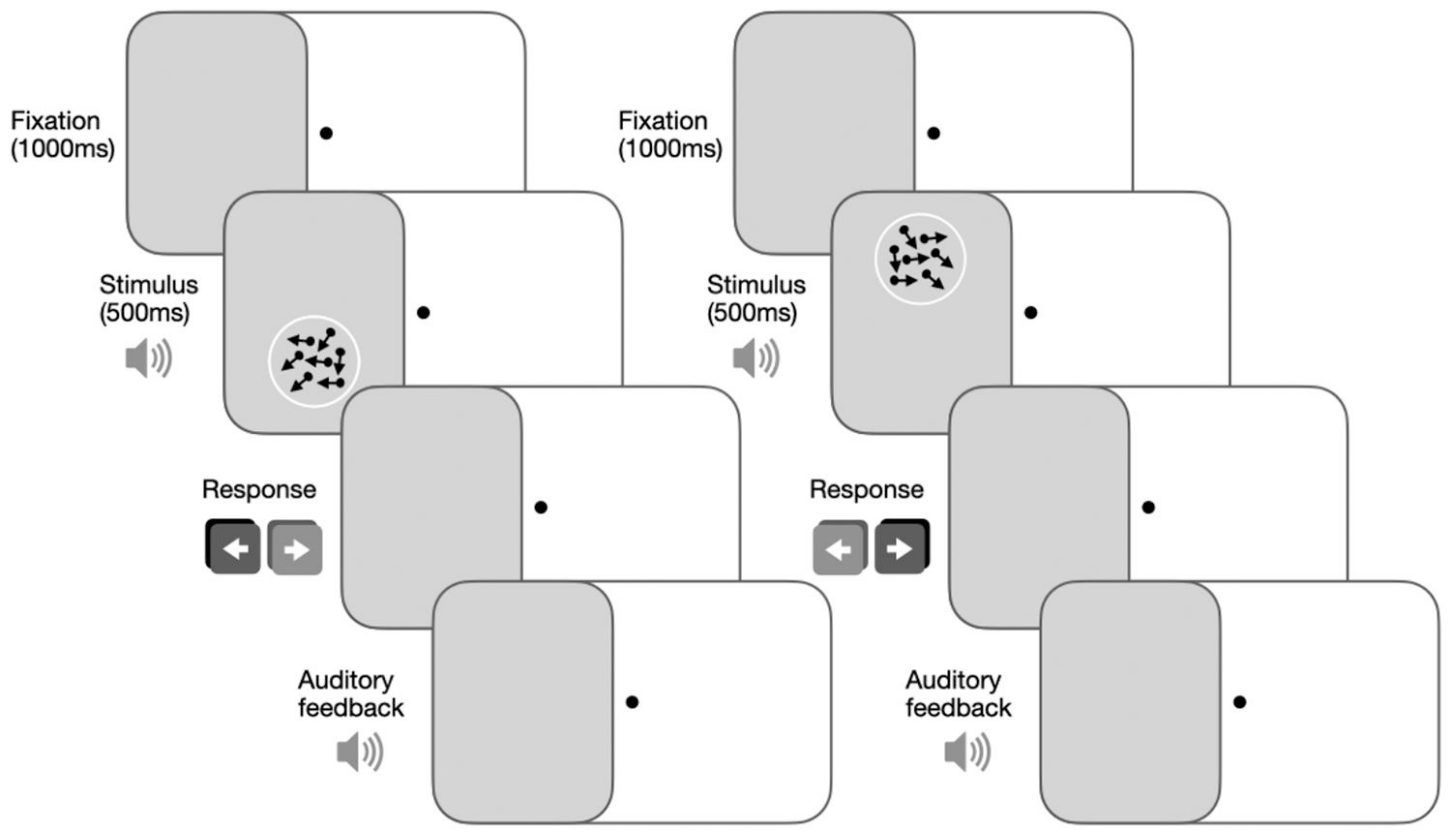
**Training paradigm.** Participants fixate centrally, and motion stimuli are presented at the training location inside the blind field. Presentation of the stimulus is accompanied by an auditory tone. Participants are asked to discriminate the global direction of movement (leftward or rightward) of the dots in the stimulus after each presentation. They are provided with auditory feedback signalling the correctness of their responses on each trial. This is repeated for 300 trials at each blind field location.

Participants will be asked to make coarse, left-right discriminations of global motion directions of random dot stimuli. Random dot stimuli will consist of black dots on a grey background (dot speed 10 degree/s, dot lifetime 250 ms, stimulus duration 500 ms; aperture 5 degrees diameter). Participant’s eyes will be positioned 42 cm away from the computer screen with a chin-forehead rest. Training difficulty will be modulated using a 3:1 staircase procedure for direction range of the dots; after three correct responses, direction range will increase from 0 to 360˚ in 40˚ steps; and after one incorrect response, it will decrease by 40˚.^5,10,11^ Auditory feedback will signal correct and incorrect responses on each trial (see Fig. 2 for schematic). Performance for each session will be calculated as a function of direction range level. A Weibull function will then be fitted to the data with a criterion threshold of 75% correct. The session threshold will be normalized to the maximum range of dot directions (360˚) to generate a normalized direction range (NDR) using the following equation:


$$\begin{array}{c} \mathrm{NDR}\;\mathrm{threshold}\;\left(\%\right)\;=(360^{\circ }\;\text{–}\mathrm{Weibull}\text{-}\mathrm{fitted}\;\\ \;\mathrm{direction}\;\mathrm{range}\;\mathrm{threshold})/360^{\circ }\;\times \;100 \end{array}$$

The initial training location for each participant will be determined by HVF perimetry at baseline using both 24-2 and 10-2 protocols. Initial training locations will be selected by measuring NDR thresholds sequentially, starting with the edge of the stimulus at the vertical meridian and moving 1˚ laterally until performance drops to chance (50% correct), and NDR thresholds are unmeasurable (designated as 100%). For all training locations, the full diameter of the stimulus will be inside the Humphrey-defined blind field border. During at-home training, as performance at each location reaches 75% correct and NDR thresholds stabilize over 5–10 consecutive sessions (less than 30% coefficient of variation), this training location will be moved to a new location 1 degree further into the blind field, along the *x*-axis (Cartesian coordinate space), and daily training will start anew.

After each at-home training session is completed, the software will automatically generate a log file detailing trial by trial performance. These log files will be emailed to the laboratory weekly by participants, allowing researchers to compute thresholds and follow their progress.

Participants will be asked to fixate centrally throughout home training. They will be reminded that fixation is essential to ensure the visual target is presented at the intended training location in their blind field. If they move their eyes, the location of the target in the brain will change, which will affect the training. Prior experience with this approach has shown that participants are highly motivated and compliant. An EyeLink 1000 Plus eye tracker (SR Research Limited, Ontario, Canada) will also be used at both research centre visits to verify fixation and ensure fixation-contingent stimulus presentation. Only after at-home training results and threshold improvements are verified in-lab with controlled fixation will specific participants be classified as having experienced visual improvement at their training locations. A failure to fixate correctly during at-home training produces anomalies in the training data that are easily detectable by researchers. If participants are looking at the stimulus rather than fixating, we will see sudden improvements in their percentage correct and NDR thresholds. Based on prior studies, percentage correct should gradually increase followed by gradual improvements in thresholds over weeks of training. Participants unable to fixate during training at home will be asked to return to the research centre for performance verification using the eye tracker. If they remain unable to fixate or cannot learn to perform the training tasks while fixating, their data will be removed from further analyses.

### Behavioural testing and MRI acquisition during pre- and post-training visits

Participants will attend the research centre for two study visits: one pre-training and one post-training (after 6 months). During these visits, motion discrimination performance will be measured with eye tracking to independently confirm the effect of training (see above for details), and participants will complete HVF perimetry and an MRI scan.

#### Humphrey visual field acquisition

Two sets of HVFs (24-2 and 10-2) will be performed monocularly with each eye using a Humphrey Field Analyser (SITA fast and SITA standard, respectively) to estimate the visual fields of each participant pre-training and post-training. Visual acuity will be corrected to 20/20, and eye tracking will be controlled to ensure central fixation during this task. This will allow us to obtain a measure of fixation stability throughout the study. Data from subjects who show fixation losses, false positives and negative errors outside of the normal range (≥20%) in either eye at any time point will be removed from further analyses. The same trained researcher will perform each assessment, which will be used to map the spatial extent of each participant’s visual field deficit.

#### Humphrey visual field data pre-processing

HVF data will be analysed as described previously.^6,12^ The 24-2 and 10-2 Humphrey software will provide the following information: (i) the deviation from the mean for each HVF testing location compared to an age-corrected normal population [pattern deviation (PD)]; (ii) the overall sensitivity difference between the tested and expected hill of vision compared to an age-corrected, normal population (perimetric mean deviation); and (iii) the 10-2 HVF will also give a test-retest variance (short term fluctuations). Given the homonymous nature of visual field deficits in this patient population, composite binocular HVF will be calculated by averaging monocular luminance detection thresholds (dB) for both eyes. Binocular 24-2 and 10-2 HVFs will then be combined at overlapping regions. Difference maps will be generated by subtracting the pre-training composite maps from the post-training composite maps. This will quantify the change in area of the HVF-defined visual deficit (defined by PD < −5 dB; deficit area) and the area of HVF where the sensitivity improved by more than 6 dB relative to pre-training (area of improvement). The area of visual field impairment will be defined as those with an impaired binocular average sensitivity of below 15 dB.

#### MRI acquisition

Scanning will use a 3T Siemens Prisma MRI scanner with a 64-channel head coil at the research centre. A structural scan will be acquired for each participant pre-training and post-training for registration and quantification of the lesion size. These will be high-resolution (1 × 1 × 1 mm^3^) whole head T_1_-weighted anatomical images (TE = 3.97 ms, TR = 1900 ms, FoV = 192 mm, flip angle = 8˚).

Diffusion-weighted data will be acquired for each subject at pre-training and post-training sessions using the UK Biobank sequence.^29^ We will use a spin-echo echo-planar imaging sequence (EPI; TR = 3600 ms, TE = 92 ms, 2 × 2 × 2) acquired in the anterior-posterior encoding direction with 50 *b* = 1000 s/mm^2^ and 50 *b* = 2000s/mm^2^ diffusion-weighted volumes acquired with 100 distinct diffusion-encoding directions and 3 × multiband acquisition. Five volumes without diffusion weighting (b0 volumes; *b*-value = 0 s/mm^2^) and three additional b0 volumes (*b*-value = 0 s/mm^2^) with posterior-anterior encoding will also be acquired to correct for image distortion.

For a subset of eight participants, a structural scan and diffusion-weighted imaging data were acquired between 2018 and 2019 using the same parameters stated here, along with a psychophysical measure of contrast detection. These data will be used as a ‘no training’ control group.

### Reproducible data management and processing

All data acquired for this project will be managed using the open science platform brainlife.io. Initially, data will be securely stored in a private project on brainlife.io and processed using state-of-the-art reproducible Apps.^30,31^ All data products will be generated by tracking their provenance (i.e. keeping a record of the combination of the generated data product and processing application used to generate the data).^30^ The data will be published publicly at the end of the project to allow other researchers to reproduce our results and reutilize our processing pipeline for future studies as described in Avesani *et al*.^30^

Reproducible, open cloud computing services planned for this study. Our pipeline will consist of 15 brainlife.io Apps (Table 1).

**Table 1:** Description, weblinks and DOI for the open cloud services used to process the data

Application	GitHub repository	Open Service DOI
FSL Anat	https://github.com/brainlife/app-fsl-anat	10.25663/brainlife.app.273
Freesurfer cortical and subcortical segmentation	https://github.com/brain-life/app-freesurfer	10.25663/bl.app.0
Tissue-type segmentation	https://github.com/brainlife/app-mrtrix3-5tt	10.25663/brainlife.app.239
Multi-Atlas Transfer Tool (MaTT)	https://github.com/faskowit/app-multiAtlasTT	10.25663/bl.app.23
FSL Topup & Eddy—CUDA	https://github.com/brainlife/app-FSLTopupEddy	10.25663/brainlife.app.287
Mrtrix3 preproc	https://github.com/brain-life/app-mrtrix3-preproc	10.25663/bl.app.68
SNR calculation	https://github.com/davhunt/app-snr_in_cc/tree/plot	10.25663/bl.app.120
FSL BET	https://github.com/brain-life/app-FSLBET	10.25663/brainlife.app.163
NODDI Fit via Amico	https://github.com/brain-life/app-noddi-amico	10.25663/brainlife.app.365
FSL DTIFIT	https://github.com/brainlife/app-fslDTIFIT	10.25663/brainlife.app.292
Fit Constrained Spherical Deconvolution Model for Tracking	https://github.com/bacaron/app-mrtrix3-act	10.25663/brainlife.app.238
Anatomically Constrained Tractography using precomputed 5tt & CSD	https://github.com/bacaron/app-mrtrix3-act	10.25663/brainlife.app.297
merge2TCKs	https://github.com/bacaron/app-mergeTCK	10.25663/brainlife.app.305
Visual white matter tracking	https://github.com/brainlife/app-trekker-roi-tracking	10.25663/brainlife.app.226
Remove tract outliers	https://github.com/brainlife/app-removeTractOutliers	10.25663/brainlife.app.195
Tract analysis profiles	https://github.com/brain-life/app-tractanalysisprofiles	10.25663/brainlife.app.361

## Planned analyses

### Behavioural changes

Based on several studies,^5-9^ we predict improvements in motion discrimination thresholds and perimetry-defined luminance sensitivity post-training compared to pre-training.

### White matter changes

We predict that after training, participants will show increases in FA in the visual white matter pathway between ipsilesional dLGN and hMT+ and ipsilesional dLGN and V1, but not between ipsilesional VPL and S1.

#### Pathways of interest

Using the brainlife.io software, we will perform analyses of specific pathways (consisting of fascicles) within the brain. The main pathway which has been implicated in residual processing in visual field deficits is between dLGN and hMT+.

We will also investigate changes in FA due to training in the pathway between dLGN and V1 and the projection between VPL and S1. The latter will act as a control tract, as it also relays sensory information between the thalamus and the cortex but is unlikely to change as a result of visual training.

#### Regions of interest

The five regions of interest (ROI; dLGN, VPL, V1, hMT+ and S1) will be obtained from anatomically defined probabilistic maps. dLGN, V1, hMT+ and S1 masks will be derived from the Glasser Atlas^32^ and VPL from the Oxford Thalamic Connectivity Probability Atlas. V1 in the damaged hemisphere will be masked by the lesion to ensure that only brain tissue is included in the definition.

To ensure that ROIs are consistent across scans and time points, ROIs will be transformed from standard space to structural space.

#### Tractography

Diffusion-weighted MRI data will be pre-processed using a series of brainlife.io Apps (see Table 1). Some of these Apps implement methods from major software libraries such as FSL,^33,34^ FreeSurfer,^35^ MRTrix,^36^ VISTASOFT and AFQ,^37,38^ LiFE and Ensemble Tractography.^39,40^ Anatomically constrained tractography and Ensemble Tractography will be used to track the visual white matter tracks of interest (e.g. brainlife.app.226 or brainlife.app.297).^37,41^

More specifically, fibre-orientation distribution function (fODF) will be estimated in each white matter voxel using constrained spherical deconvolution (CSD).^42^ Fascicle tracking will be performed on these fODFs using a probabilistic ‘region to region’ algorithm implemented in MRtrix.^36^ A union mask will be created by combining the two ROIs. Streamlines will then be generated from 10 000 seeds inside this mask. Streamlines will only be included if they touch both ROIs and travel within white matter. The total number of streamlines will be constrained to 1 000 000. A curvature radius threshold of 1 mm will be used and a step size of 0.2 mm.

An anatomically informed approach will be used to identify core fascicles.^19,37-39,43,44^ Outlier fascicles will then be removed from analyses so that the tract estimate is conservative. Outlier fascicles will be defined as those located more than 2.6 standard deviations away from the core of the tract or 2.8 standard deviations longer than the mean tract length, using a Gaussian distribution to represent fascicle length and distance. If this calculation is not possible because there are a small number of sparse fascicles (<10), then it will be assumed that tracking was not possible between two areas of interest. Tracts will then be processed using brainlife.io.^30^ Pathways of interest will be defined anatomically as tracts that pass between the two ROIs in the same hemisphere.

#### White matter microstructure

We plan to use both diffusion tensor imaging (DTI; brainlife.app.292) and neurite orientation dispersion and density imaging (NODDI; brainlife.app.35)^45^ models to estimate the microstructural properties of the white matter tracts at each time point.

We will then combine the microstructural parameters estimated via either DTI (FA and MD) or NODDI [neurite density index (NDI), orientation dispersion index (ODI) and isotropic volume fraction (ISOVF)] with the spatial information of each tract trajectory. This will give us an average of each parameter weighted by distance from the mean of the tract at each trajectory. Each tract will be resampled to 100 nodes, distributed equally along the length of the tract.^38^ The initial and last 15 nodes (1–15 and 85–100) will be removed to limit grey matter contamination and partial volume effects. These clipped profiles will then be used to calculate measures of mean tract microstructural parameters. These tract profiles will be used to determine the white matter microstructure parameter for each participant and time point along pathways between dLGN and hMT+, dLGN and V1 and VPL and S1. This will be calculated along the core of the pathways to reduce partial volume artefacts.

To ensure consistency across repeated scans, the mean FA will be calculated across the brain (with the lesion masked out) for each FA image and used to normalize the FA extracted from the individual pathways.

### Planned statistical analyses

#### Control analyses

Our hypotheses are based on the assumption that FA will increase with visual training. We will therefore use positive control analyses to confirm that FA does not change without training. In a subset of eight participants, we have an earlier dataset (acquired between 2018 and 2019) which we will compare to baseline as a ‘no training’ control. We will use three repeated measures *t*-tests to determine whether there is a significant change in FA between the initial scan and baseline scan in the three tracts of interest. We predict that there will be no increase in FA between the initial scan and baseline in the LGN-hMT+ or LGN-V1 pathways in these participants.

#### White matter and recovery

Linear regression analyses will be used to determine the relationship between motion discrimination thresholds (Hypothesis 1) and area of improvement on the HVFs (Hypothesis 2) and changes in FA in the three tracts (ipsilesional dLGN-hMT+, dLGN-V1 and VPL-S1). These will be implemented in R.^26^ We hypothesize that there will be a significant positive relationship between (i) improvement in motion discrimination thresholds and increases in FA in the dLGN-hMT+ tract (Hypothesis 1; one-tailed) and (ii) area of improvement on the HVF perimetry and increase in ipsilesional FA in the dLGN-V1 tract, after training (Hypothesis 2; one-tailed). We predict there will be no relationship between change in FA in the ipsilesional VPL-S1 and behavioural measures due to training.

#### Biomarkers of recovery

A linear regression will be used to determine whether pre-training FA in the ipsilesional dLGN-hMT+ predicts improvement in motion discrimination thresholds. We predict a significant positive relationship between pre-training FA in the dLGN-hMT+ tract and improvement in motion discrimination thresholds (Hypothesis 3; one-tailed), a finding absent in dLGN-V1 and VPL-S1 pathways.

### Exploratory analyses

#### Alternative white matter metrics

Due to our sample size and power calculation, we are only powered to detect large effect sizes across a small number of tests. We have therefore limited the number of hypotheses that are directional and registered to those based strongly on the literature. However, in addition to the planned analyses described above, we will also explore alternative DTI (MD, RD and AD) and NODDI (NDI, ODI and ISOVF) white matter metrics in relation to improvements in behaviour (motion discrimination; HVFs). This will allow us to verify and explore any changes we find in FA. Linear regression analyses will be used to explore the relationship between behaviour and these white matter metrics. These will be corrected for multiple comparisons using Bonferroni correction, i.e. *P* < 0.05/6 = <0.008.

#### Alternative pathways

Alternative pathways have also been suggested to be involved in blindsight, such as the superior colliculus/pulvinar pathway to hMT+ and ipsilateral hMT+ to contralateral hMT+. We will also aim to explore the relationship between these pathways and improvements in behavioural measures using linear regression analyses. These will also be corrected for multiple comparisons using Bonferroni correction.

## Data Availability

We confirm that on in-principal acceptance, we will register an approved protocol on the Open Science Framework. We will also make openly available all appropriately anonymized raw data supporting the reported analyses, analysis code and laboratory log from this study.
